# Non-customized data asset evaluation based on knowledge graph and value entropy

**DOI:** 10.1371/journal.pone.0316241

**Published:** 2025-03-18

**Authors:** Wei Zhang, Yan Gong, Zhinan Li, Yuefeng Xu

**Affiliations:** 1 Institute of Science and Technology Information, Beijing Academy of Science and Technology, Beijing, China; 2 Faculty of Arts and Social Science, The University of Sydney, Sydney, New South Wales, Australia; Shri Ramswaroop Memorial University, INDIA

## Abstract

With the rapid expansion of non-customized data assets, developing reliable and objective methods for their valuation has become essential. However, current evaluation techniques often face challenges such as incomplete indicator systems and an over-reliance on subjective judgment. To address these issues, this study presents a structured framework comprising 17 key indicators for assessing data asset value. A neural network is employed to calculate indicator weights, which reduces subjectivity and enhances the accuracy of the assessment. Additionally, knowledge graph techniques are used to organize and visualize relationships among the indicators, providing a comprehensive evaluation view. The proposed model combines information entropy and the TOPSIS method to refine asset valuation by integrating indicator weights and performance metrics. To validate the model, it is applied to two datasets: Bitcoin market data from the past seven years and BYD stock data. The Bitcoin dataset demonstrates the model’s capability to capture market trends and assess purchasing potential, while the BYD stock dataset highlights its adaptability across diverse financial assets. The successful application of these cases confirms the model’s effectiveness in supporting data-driven asset management and pricing. This framework provides a systematic methodology for data asset valuation, offering significant theoretical and practical implications for asset pricing and management.

## 1. Introduction

### 1.1 Background

With the rapid development of the digital economy, data has become one of the most core assets of modern businesses and organizations [1]. More and more organizations and enterprises realize the value of data and want to use data to optimize business operations, provide personalized products and services, and even strategic planning [2]. Among them, Non-customized Data Assets (NCDAs), as a type of data assets, occupy an important position in enterprise data management due to their versatility and wide applicability. Non-customized data usually refers to general-purpose data that is not tailored to the needs of a specific user, and is widely used in a variety of fields, such as market forecasting, data analytics and business intelligence [3].

In the existing studies, the valuation of data assets has achieved great progress, such as exploring the evolution of the concept of data assets [4,5], clarifying the importance of data assets [6], and valuing data assets [1]. Traditional data asset management and evaluation focuses more on structured data and has a higher degree of standardization. However, for non-customized data assets, the existing management and evaluation system is not applicable because of its large scale, unclear value and uneven quality. How to collect and process these massive non-customized data, explore its commercial value, and find a suitable evaluation method has become an urgent problem to be solved. With the rise of data-driven decision-making, enterprises are increasingly demanding the use of non-customized data to improve management and innovative products and services. However, the management and application of these data face many challenges. How to build an automated non-customized data asset evaluation and management system to continuously track the value of data and support enterprises to make correct decisions is one of the key objectives of current research. The main problem is assessing the value of data assets while taking into account the uncertainty of non-customized data assets.

Based on a comprehensive analysis of the unique characteristics of non-customized data assets, the practical needs of enterprises, and the limitations identified in existing research, this study proposes a novel evaluation framework aimed at addressing these gaps. The framework includes a systematic process of collecting large-scale non-customized data, improving its quality, and constructing a robust assessment indicator system. This allows for a more accurate, quantitative, and dynamic evaluation of the value of such data assets. By employing advanced methods, including a neural network-based approach for indicator weighting and an information entropy-TOPSIS model, this study provides a practical solution for enterprises seeking to optimize their use of non-customized data assets. The findings are expected to offer valuable insights for industries aiming to leverage these assets more effectively, ultimately enhancing data-driven innovation, promoting sustainable development, and fostering broader economic growth and social advancements.

### 1.2 Literature review

#### 1.2.1 Factors affecting the valuation of data assets.

With the continuous advancement of informatization and digital transformation, enterprises possess and control an explosive growth of diverse data assets in both type and quantity, accumulating at a geometric rate. Various data assets are influenced and constrained by many factors, such as technological innovations, market demands, regulatory policies, etc. Dong and Zhang studied the impact of data assets on data assets, and the quality of data assets has become a concern and worthy of research [7]. Aremu et al. studied the impact of the life cycle of assets on data assets. He believed that the life cycle of monitoring systems can provide current diagnosis, prediction, and information that can guide maintenance decisions [8]. Data standardization is essential for preserving and exchanging scientific knowledge, and it requires appropriate data formats and sources [9]. Braga and Andrade proposed a data-driven asset degradation maintenance decision support system and explored the relationship between data governance and data assets [10]. In addition to the assets themselves, there are several external factors that affect data assets. The social context has a strong impact on data assets, and Taera et al. studied the volatility and persistence of external shocks in financial and alternative asset markets during the crisis triggered by COVID-19 and the war in Ukraine [11]. In addition, social policies will also have a great impact on data assets. Liu et al. explored the impact of China’s aging family population and pension insurance on household financial asset allocation [12]. Analyzing and evaluating quality risk is critical to leveraging the value of data assets, and You et al. proposed a proactive assessment framework for data asset quality risk based on improved FMEA [13].

Furthermore, in addition to the impact of internal and external factors, the management of upstream and downstream assets also affects the valuation of data assets. Noshahri et al. predictive maintenance planning and assessment of asset condition by examining data-driven management of sewer assets [14]. It is very important to establish an accurate and comprehensive data asset evaluation framework for enterprises to evaluate the value of data assets and account processing [15]. A scientific and systematic evaluation framework can help enterprises accurately judge the intrinsic value of different types of data assets, provide a basis for data asset management decisions, achieve standardized monitoring and accounting of data assets, and better reflect the contribution of data assets in corporate performance. Qu et al. examined the asymmetric spillover effects of Bitcoin to green and traditional assets by using a complete allocation framework established by the recently developed quantile-to-quantile approach [16].

#### 1.2.2 Evaluation method.

Systematic evaluation and valuation of enterprise data assets is a key part to maximize the value of data assets. Wang and Zhao combed the research results of domestic and foreign scholars on the valuation of data assets and classified the valuation methods of data assets into four categories [17]. The establishment of a scientific evaluation system can objectively calculate the flow value and potential value of data assets, and provide a basis for enterprises’ data asset investment, operation and management decisions [18]. Through the continuously optimized data asset evaluation mechanism, enterprises can continuously realize the value transformation of data assets, and make data truly become the production factor and core capital of enterprises. Matevž Skočir et al. explored the significance of multi-factor asset pricing models in business valuation, centred around the eight-factor model [19]. TOPSIS based model is also applied to the evaluation of data assets, Dong ang Zhang designed Fermatean Fuzzy TOPSIS model based on TOPSIS model to solve the problem of Commercial Bank Data Asset Quality Evaluation [7]. Yang et al. used the maximum correlation portfolio (MC) method to test the asset pricing model [20]. Manresa et al. estimated the identified set of SDFS and the price of risk that are compatible with the asset pricing constraints of a given model [21]. Wu and Zhang solved the pricing problem of data assets based on Based on the Real Option Method [22]. For asset valuations, Koo and Muslu compared the flow of funds and asset valuations of bond mutual funds and found that bond mutual fund managers overstate the value of their assets if they also manage a portfolio based on performance fees [23]. Lin et al. has developed a framework for evaluating the net present value (NPV) of geological hydrogen storage that integrates the latest technical and economic analysis and market operation, using capital asset pricing model (CAPM) and relevant financial theory scheduling knowledge [24]. Lu and Yang proposed a new European option pricing formula based on the underlying stock, where the price is determined by demand and supply in a given transaction [25]. For the existing models, we summarized and pointed out the shortcomings of the existing models, as shown in Table 1.

**Table 1:** Aggregation of existing models.

Reference	Model	Methodology applied	Shortcomings
Li et al., 2024	DE0A-BCC Model	Construction of the indicator system	Insufficient consideration of influencing factors
Skočir and Lončarski, 2024	multi-factor asset pricing model	around an eight-factor model	Calculation of coefficients based on regression is too simple and not comprehensive enough
Yang et al., 2023	the method of Maximum-Correlated (MC) Portfolios	Q-statistics and Sharpe ratios	Only non-traded factors were considered
Manresa et al., 2023	econometric methodology	Based on economics	Only comparisons with several common types of models are presented, and the advantages over other models are uncertain
Dong et al, 2023	Fermatean Fuzzy TOPSIS	Improvement based on TOPSIS	Calculation of evaluation indicators is too subjective and links between factors are not clearly established
Feng et al., 2024	Valuation after construction of evaluation index system by AHP method	Calculated based on AHP method	Calculation of indicator weights is too subjective
Jia-qi et al., 2023	Real Option Method of intangible assets	Based on the Real Option Method	Incomplete consideration of influencing factors

#### 1.2.3 Knowledge graph.

In recent years, knowledge graphs have been widely used in many fields. By building a network of relationships between entities, knowledge graphs can empower smarter applications. Knowledge graph has been widely used in art field [26], transportation field [27], civil engineering [28], mechanical engineering [29], power engineering [30].

For the impact of different factors on data assets, most scholars have only explored the impact of one factor on data assets or established an incomplete indicator system. In addition, most of the existing research is on customized data assets, and there is limited research on non-customized data assets. Most of the assessment methods that currently exist use human scoring, which is more subjective [31]. Therefore, we construct an evaluation index system and model for non-customized data assets to fill this research gap. We establish a complete indicator system with seventeen indicators, including internal factors, external factors, upstream and downstream data assets, and the interaction between different factors. In addition, we combine the constructed index system with knowledge graph that meets the assessment of non-customized assets. On this basis, we use the neural network method to determine the weights of indicators, and combine the information entropy and the TOPSIS model to establish the evaluation model of non-customized data assets.

### 1.3 The main contribution of this study

This study aims to address subjectivity and incompleteness in evaluating non-customized data assets. To achieve this, a comprehensive index system is constructed, a neural network is used to calculate objective index weights, and a knowledge map is built to capture intrinsic relationships among indices. Based on these components, an integrated evaluation model combining information entropy and TOPSIS is developed to quantify non-customized data asset values. The proposed framework covers key dimensions of non-customized data assets, reduces subjective bias, and enhances evaluation objectivity and accuracy. This framework provides an innovative approach to evaluating complex, unstructured data assets by integrating multiple objective methodologies. Quantifying and visualizing the evaluation process is expected to facilitate data-driven decisions and provide new insights for managing non-customized data assets.

- A new comprehensive index system is built to evaluate non-customized data assets. This study constructs an index system with 17 carefully selected indicators, covering key dimensions of asset properties, market value, and corporate performance. A knowledge graph captures the intrinsic relationships among indicators, further enhancing the system’s completeness. Compared to existing methods, this index system provides a more comprehensive, objective assessment of the multidimensional value of non-customized data assets. This systematic, knowledge-based index framework fills gaps in current evaluation practices and improves quantification in this complex domain.
- A neural network calculates objective index weights, overcoming subjectivity in traditional methods. This study leverages neural networks for adaptive learning, nonlinear modeling, high-dimensional data processing, and flexibility. This neural network-based approach objectively determines weights for each index, minimizing subjective biases. Replacing manual weighting with neural network calculations enhances objectivity and accuracy in overall evaluation. This innovative technique addresses the longstanding issue of subjectivity in data asset assessment, representing a major improvement over conventional method.
- An integrated evaluation model combining information entropy, TOPSIS, and neural networks is developed. Using neural network-derived index weights, this study proposes a hybrid model integrating information entropy and TOPSIS methods. This unified approach leverages the strengths of entropy weighting and TOPSIS multi-criteria decision-making. Neural networks’ nonlinear modeling capabilities further enhance the model’s adaptability to complex, real-world problems. Compared to conventional linear models, this integrated approach captures nonlinear relationships, enabling more accurate and reliable data asset evaluation.
- The framework and model were validated using Bitcoin data and BYD stock data to demonstrate its accuracy and practicality. The findings further demonstrate the validity of the methodology and provide new theoretical and practical support for the valuation of non-customized data assets. The study is structured as follows

The structure of this study is as follows. Section 2 presents the evaluation index system for non-customized assets and describes the research framework. Section 3 introduces the models, including the neural network model, knowledge graph, and information entropy-TOPSIS model. Section 4 uses Bitcoin data and BYD stock data for demonstration, while Section 5 summarizes the study’s findings.

## 2. Study framework

### 2.1 Indicators’ introduction

Non-customized data assets are general-purpose data that are not specifically tailored. Their main characteristics include broad sourcing, general content, ease of access and use, wide coverage, low usage costs, and minimal development and maintenance expenses. Given the complexity of non-customized data, this study examines impact indicators from four primary categories, each containing specific secondary indicators. The details are as follows:

#### 1) Intrinsic factors affecting the value of non-customized data assets.

Data source: This indicator takes into account the impact of the type of data channel, channel quality, and real-time performance.

Data scale: This indicator considers the size of the data volume and the impact of the comprehensive scheduling of the spatiotemporal dimension.

Data format: This metric takes into account how easy it is to convert structured versus unstructured data.

Data quality: This metric takes into account the impact of data accuracy, completeness, consistency, and reliability.

Value mining: This metric takes into account the impact of data’s potential for insight generation and application.

Data governance: This indicator takes into account the impact of data management mechanisms and data security measures.

Attribution of assets: This indicator takes into account the impact of clear intellectual property rights on the data.

#### 2) External environmental factors.

Technological development: This indicator takes into account the impact of relevant technological advances that have enhanced the value of data and how it is used.

User demand: This metric takes into account the dependence of different users on relevant data and the impact of application innovation.

Data Ecology: This indicator takes into account the impact of an open and shared environment that facilitates the mining and release of data value.

Regulatory environment: This indicator takes into account the impact of data security and privacy requirements in relevant industries.

Market development: This indicator takes into account the impact of the overall level of data production and application in the industry.

Social cognition: This indicator takes into account the impact of trust and acceptance of new technologies and data applications.

#### 3) Upstream and downstream assets.

Upstream source data: This metric takes into account that an increase in the value of source data assets benefits derived data value.

Downstream applications: This metric takes into account how data plays a role in a wider range of downstream applications.

#### 4) Interactions between different asset classes.

Other non-custom assets: Considering the interconnected non-custom assets, changes in their value will affect each other.

Custom Assets: Consider that custom asset applications rely on non-custom data for data sources and analytics insights.

**Fig 1:**
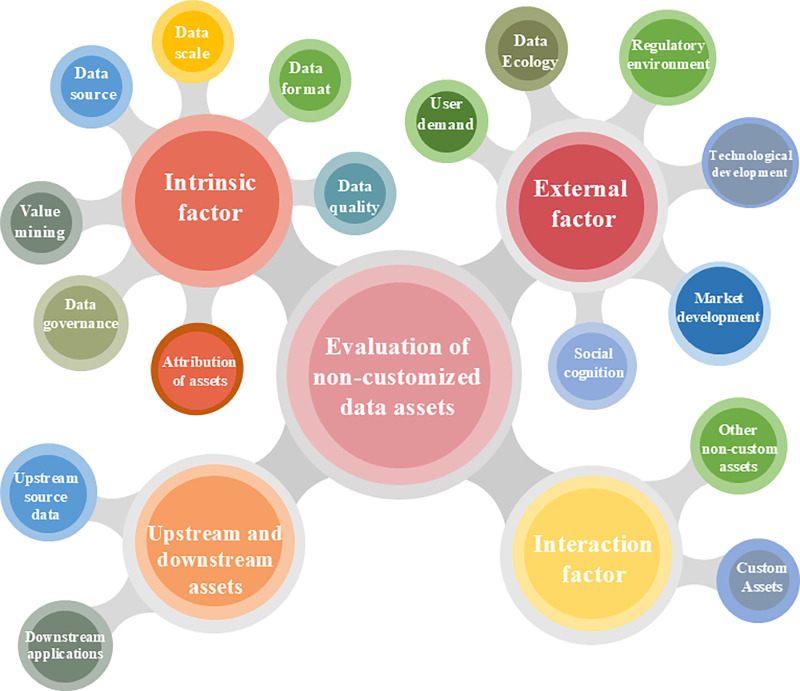
The initial knowledge graph.

The initial knowledge graph is shown in Fig 1.

### 2.2 Evaluation framework

**Fig 2:**
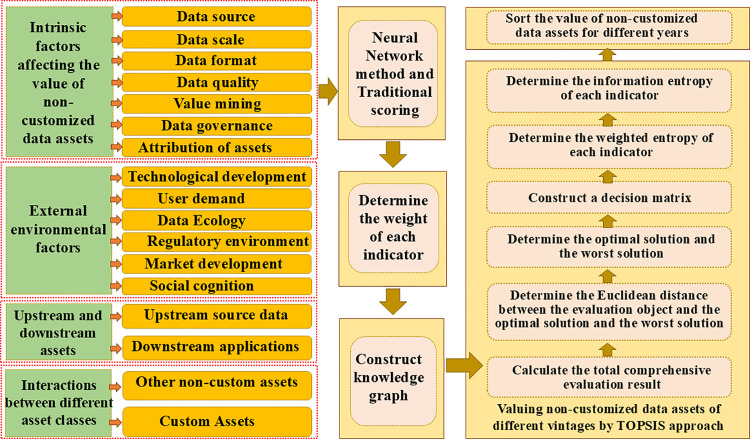
The Evaluation framework.

Non-customized assets are unique and irreplaceable, characterized by their non-substitutability. Based on extensive literature review and the unique characteristics of non-customized data assets, this study identified four primary indicators and 17 secondary indicators to establish an evaluation system for non-customized data assets. This system comprehensively accounts for internal factors, external influences, upstream and downstream assets, and interactions among different asset types. Using this established indicator system, the study applies a neural network method to assign weights to each indicator. Subsequently, the study integrates the information entropy model with TOPSIS to develop a comprehensive evaluation model. The model then calculates a total evaluation score at a specified assessment stage, determining the value of the non-customized data asset based on this structured approach. The framework structure is illustrated in Fig 2.

## 3. Modeling

This section consists of three parts: Neural network modeling, Constructing knowledge graph and Neural network-based information Entropy-TOTSIS model. Table 2 shows some simple concepts of the method we used [32–36].

**Table 2:** Relevant terms .

Term/Concept	Explanation
Neural Network	A computational model used in this study to predict the weights of indicators. The network learns from historical data and calculates the relative importance of each indicator based on training data.
Hidden Layer Activation Function (Tansig)	The Tansig (hyperbolic tangent sigmoid) activation function is used in the hidden layer, mapping values to a range between -1 and 1. This allows the model to capture complex data patterns with greater sensitivity, which is critical for accurate indicator weight prediction.
Output Layer Activation Function (Purelin)	The Purelin (linear) activation function is applied in the output layer to produce continuous, unbounded outputs. This function is suitable for regression tasks, allowing the model to generate interpretable output values for data asset evaluation.
Loss Function (Mean Squared Error, MSE)	The Mean Squared Error (MSE) loss function is used to measure the difference between predicted and actual values, penalizing larger errors more heavily. This choice improves the model’s accuracy in predicting weights by prioritizing minimization of large deviations.
Knowledge Graph	A tool used in this study to visually represent the relationships between different indicators. It organizes the indicators into a structured format, helping to reveal how each indicator influences others.
Nodes	Represent the individual indicators in the knowledge graph. Each node corresponds to a specific feature or characteristic used in the evaluation model (e.g., price volatility, trading volume).
Edges	The connections between nodes in the knowledge graph, representing the relationships or dependencies between indicators. These edges illustrate how different indicators influence one another.
Information Entropy	A statistical measure used to quantify the uncertainty or diversity in a set of indicators. In this study, entropy is used to assess the distribution of values across indicators to determine their relative importance.
Information Entropy Weight	The method used to assign weights to each indicator based on its entropy. Indicators with lower entropy (less variation) are assigned higher weights, as they contribute more decisively to the evaluation. Conversely, indicators with higher entropy (more variation) are given lower weights. This ensures that more stable indicators have greater influence in the model.
TOPSIS (Technique for Order Preference by Similarity to Ideal Solution)	A decision-making method applied in this study to rank different time periods based on their proximity to the ideal solution. TOPSIS ranks alternatives based on the distance from the ideal (best) and negative ideal (worst) solutions.
Ideal Solution	Represents the best possible values for all indicators, where each indicator is at its maximum level. It serves as a reference point to compare the evaluated periods or alternatives.
Negative Ideal Solution	Represents the worst possible values for all indicators, where each indicator is at its minimum level. It is used to measure how far each evaluated period deviates from the worst-case scenario.
Distance Measure (Euclidean or Similarity)	In TOPSIS, this measure calculates the distance between each alternative and the ideal and negative ideal solutions. The alternative with the smallest distance to the ideal solution is considered the best.

### 3.1 Neural network modeling

This study utilizes a multiplayer perceptron (MLP) neural network to determine indicator weights, overcoming limitations of traditional weighting approaches. Unlike manual assignment of weights in conventional models, the MLP enables automated learning and optimization of weights based on the input data [37]. This allows more accurate capturing of relationships between indicators and improved decision-making accuracy. Additionally, the nonlinear modeling capacity of neural networks can handle the complex interactions and patterns among indicators of different types and characteristics. By contrast, traditional linear models cannot fully account for these multidimensional relationships. Moreover, neural networks can process high-dimensional data and extract salient information, adapting well to real-world complexity. Their flexibility and scalability also allows easy adjustment for new decision scenarios and indicators. In summary, the data-driven MLP neural network approach provides adaptive, nonlinear and robust weight calculation. By replacing subjective expert weighting with automated neural network learning, this method delivers more reasonable and reliable indicator weights for evaluating multifaceted non-customized data assets.

#### 3.1.1 Modeling steps.

***Step 1*: Initialize Weights**

Initialize the weight matrices that connect the input layer to the hidden layer and the hidden layer to the output layer based on the network structure. The network structure is as follows:

Input Layer 17 neurons, representing the 17 indicators' scores for each year.

Hidden Layer A single hidden layer with 10 neurons.

Output Layer 17 neurons, representing the predicted weight for each indicator.

***Step 2*: Forward Propagation**

Pass input samples (the indicator scores for each year) through the network, computing each layer’s output in turn.

Data flows from the input layer through the hidden layer to the output layer:

If we denote the input data as *X* (of size 6 × 17), then the input is multiplied by the weight matrix $W_{1}$ from the input to the hidden layer, and then passed through an activation function to get the hidden layer output. The hidden layer output is then multiplied by $W_{2}$, the weights between the hidden layer and output layer.

***Step 3*: Activation Function**

Apply an activation function to introduce non-linearity.

Hidden Layer Activation Function (Tansig): Uses the Tansig (hyperbolic tangent sigmoid) activation function, as shown in Eq. (1).


$$\begin{array}{c} \begin{array}{l} f\left(x\right)=\frac{2}{1+e^{-2x}}-1 \end{array} \end{array}$$ (1)

where *x* is the input to the activation function. This maps the values to a range between -1 and 1, allowing for greater sensitivity in capturing data patterns.

Output Layer Activation Function (Purelin): Uses the linear activation function (Purelin) to maintain continuity in the output, suitable for regression tasks. The output layer uses a linear activation function (Purelin), expressed as Eq. (2).


$$\begin{array}{c} f\left(x\right)=x \end{array}$$ (2)

***Step 4*: Loss Function**

The loss function measures the difference between the predicted outputs and the actual target values. Here, the code uses Mean Squared Error (MSE) as the default loss function, as shown in Eq. (3).


$$\begin{array}{c} MSE=\frac{1}{N}\overset{N}{\underset{i=1}{\sum }}{\left(y_{true}-y_{pred}\right)}^{2} \end{array}$$ (3)

where $y_{true}$ is the actual value, $y_{pred}$ is the predicted value, and *N* is the number of samples. MSE penalizes larger errors more significantly, which helps improve the accuracy of the model.

***Step 5*: Backpropagation**

Backpropagation calculates the gradient of the loss function with respect to each weight by propagating the error backward through the network.

Gradients guide adjustments to each layer’s weights, reducing the overall error in future predictions.

***Step 6*: Weight update**

Update the weight matrices using an optimization algorithm. In MATLAB, the train function typically uses the Levenberg-Marquardt algorithm, a powerful method for improving convergence speed, especially for non-linear problems.

Weight update formula for gradient descent, as shown in Eq. (4).


$$\begin{array}{c} W=W-\vartheta \;\cdot \;\nabla W \end{array}$$ (4)

where *W* is the weight matrix, *ϑ* is the learning rate, and ∇W represents the gradient of the loss function with respect to *W*.

***Step 7:* Repeat Steps 2-6**

Repeat ***Steps 2-6*** until the model reaches a stopping criterion, such as a maximum number of iterations or convergence of the loss function to a predefined threshold.

#### 3.1.2 Rules for determining the number of neurons in the hidden layer.

The number of neurons in the hidden layer is determined by the following rule.

Hidden layer neurons should be greater than half the sum of the input and output neurons, as shown in Eq. (5).


$$\begin{array}{c} HiddenNeurons>\frac{InputNeurons+OutputNeurons}{2} \end{array}$$ (5)

Here, both the input and output layers contain 17 neurons, resulting in a hidden layer with 10 neurons, which meets this criterion.

### 3.2 Constructing knowledge graph

In this study, four first-level indicators and 17 second-level indicators were selected to construct the influencing factors of non-customized data assets. A knowledge graph was developed utilizing relevant insights from complex networks. The decision to employ knowledge graphs is particularly justified by their unique ability to reveal hidden relationships within complex datasets, which is critical for the accurate assessment of non-customized data assets in this research context. Knowledge graphs provide a structured representation of data, allowing for the identification of interdependencies and correlations among various indicators. This capability is essential for uncovering insights that traditional evaluation methods may overlook, thus ensuring a more comprehensive understanding of the factors influencing non-customized data assets. Furthermore, the knowledge graph facilitates the analysis of indicator interconnectivity, enabling the identification of pertinent weight distributions across the 17 indicators. This interconnectivity analysis is pivotal in assessing how various factors interact and influence non-customized data assets. Specific applications of knowledge graphs within the domains of data governance and management have been cited to illustrate their efficacy in uncovering underlying data relationships, thereby enhancing the objectivity and robustness of the evaluation process.

In the context of this study, the knowledge graph serves as a foundational tool for analyzing the selected indicators. Node degree and node number were utilized to ascertain their graph distribution, and Pajek software was employed for the construction of the knowledge graph. Node degree, a fundamental parameter characterizing the properties of nodes within complex networks, is calculated as shown in Eq. (6).


$$\begin{array}{c} deg\left(i\right)=\underset{j\in n}{\sum }a_{ij} \end{array}$$ (6)

where $\text{deg}\left(i\right)$ is the node degree of node *i* and $a_{ij}$ is the side where node *i* and *j* are connected. The node betweenness is calculated as shown in Eq. (7). The node betweenness is the ratio of the number of shortest paths passing through a node *k* to the number of all shortest paths in the network.


Beti=∑j,k∈n,j≠kϱjiiϱji (7)

where $Bet\left(i\right)$ is the number of nodes *i* and $\varrho_{ji}$ is the sum of all shortest paths in the network.

### 3.3 Neural network-based information Entropy-TOPSIS model

This study combines the information entropy and TOPSIS model constructed for calculating non-customized data assets. The information entropy-TOPSIS model is a multi-indicator decision analysis method that integrally considers the information entropy of indicators and the similarity between indicator values. The advantages of this model are presented in the following four aspects.

1) Considering the relationship between the information entropy of the indicators and the indicator values comprehensively, improves the sensitivity of the evaluation model to the comprehensive performance of the indicators.
2) Ensuring that the importance of the different indicators is reasonably taken into account by determining the weights of the indicators and performing the normalization process, as well as eliminating the differences in the magnitude of the indicators and the bias of the weights between the indicators.
3) Having results with strong interpretability, enables the decision-makers to better understand the evaluation results and make decisions.
4) flexible adjustment and expansion, which can adjust the weights and introduce other evaluation indicators or methods according to actual needs.

This study is based on calculating the weights of the indicators with neural networks and then utilizing the constructed information-entropy-topics model. Compared with the traditional model, the model has the ability of adaptive weight calculation, which can accurately capture the importance and comprehensive performance of indicators. At the same time, the model is able to handle nonlinear relationships and high-dimensional data with flexibility and scalability. Its advantage lies in providing reliable decision support, providing comprehensive and accurate information to decision-makers, and helping to optimize the decision results. This model has important application prospects in the decision-making process of complex problems and provides a reliable tool for decision-makers.

#### 3.3.1 Fundamental assumption.

1. **Assumption of correlation between indicators.**

The model assumes that there is some correlation between different indicators, i.e., that they provide relevant information on the object of evaluation. This correlation can be quantified by calculating the similarity or correlation coefficient between indicators. Correlation helps to identify redundant information and ensure the validity of different indicators in a comprehensive evaluation [38].

2. **Assumption of the indicator values can be expressed through weights.**

The model assumes that the importance of different indicators for the evaluation object is different and can be expressed by assigning weights. These weights reflect the decision maker’s preference and the importance of each indicator. The use of weights to express the importance of indicators is a common practice in MCDM. Objective weights determined by methods such as entropy can reflect the importance of information among indicators, and this method is widely used for decision analysis in economics and management [39].

3. **Assumption of the indicator values can be normalized.**

Normalization is a necessary step to ensure that indicators from different units can be evaluated on the same scale [40]. In order to eliminate the differences in scale between indicators, the model assumes that the indicator values can be normalized and transformed into relative scale. This ensures that different indicators are comparable in the comprehensive evaluation.

4. **Assumption of the best solution is the one with the smallest distance between the ideal solution and the negative ideal solution.**

The model assumes that the best solution is the one with the smallest distance between it and the ideal solution (with the maximum value) and the largest distance between it and the negative ideal solution (with the minimum value). This allows the best solution to be determined by calculating the distance between the solution and the ideal and negative ideal solutions. This assumption is at the heart of the TOPSIS methodology and ensures that the distance measures of ideal and negative ideal solutions are effective in helping decision makers to identify the best solution [41].

#### 3.3.2 Information-Entropy-TOPSIS evaluation model based on MLP.

There are m evaluated objects and each evaluated object *n* evaluation indicators. $a_{ij}$ is the evaluation indicator of the *j* indicator and the *i* object. The initial judgment matrix is constructed.

***Step 1*:** Calculate the information entropy value of the *j* indicator in the non-customized data evaluation indicator system.

The formula for calculating the entropy value of the indicator is shown in Eq. (8).


$$\begin{array}{c} H_{j}=-k\overset{m}{\underset{i=1}{\sum }}p_{ij}\text{ln}\left(p_{ij}\right)\left(j=1,2,\cdots ,n\right) \end{array}$$ (8)

where $k=1/\text{ln}\left(m\right)$ is the information entropy coefficient, $H_{j}$ is the information entropy of each indicator, $p_{ij}$ is the weight matrix calculated by the method of 3.1. $p_{ij}=d_{ij}/\overset{m}{\underset{i=1}{\sum }}d_{ij}$, $p_{ij}\in \left[0,1\right]$ is the weight of the *j* indicator of the *i* evaluation object. In order to avoid the situation of $p_{ij}$, when $p_{ij}=0$ and $p_{ij}\approx \varepsilon$ (where *ε* is an infinitesimal non-zero value). This adjustment ensures that logarithmic calculations are stable without significantly impacting the weight accuracy.

***Step 2*:** Calculate the weighted entropy of each indicator, as shown in Eq. (9).

$$\begin{array}{c} W_{j}\text{=}\frac{1-H_{j}}{\sum_{j=1}^{n}\left(1-H_{j}\right)}\left(j=1,2,\cdots ,n\right) \end{array}$$ (9)

where $W_{j}$ is the weight entropy of each indicator, $W_{j}\in \left[0,1\right]$, $\overset{\text{n}}{\underset{j=1}{\sum }}W_{j}=1$.

***Step 3*:** Construct a decision matrix. The normalized indicator value and the weighted entropy of the indicator are combined into a decision matrix, as shown in Eq. (10).

$$\begin{array}{c} r_{ij}=W_{j}p_{ij}\left(i=1,2,\cdots ,m;j=1,2,\cdots ,n\right) \end{array}$$ (10)

***Step 4*:** Determine the optimal solution $Q_{i}^{+}=\left(i=,2,\cdots ,m\right)$ and the worst solution $Q_{i}^{-}=\left(i=1,2,\cdots ,m\right)$ of the evaluation object, and the specific calculation methods are shown in Eq.s (11) and (12).

$$\begin{array}{c} Q_{i}^{+}\text{=}max\left\{r_{i1},r_{i1},\cdots ,r_{in}\right\} \end{array}$$ (11)

$$\begin{array}{c} Q_{i}^{-}\text{=}min\left\{r_{i1},r_{i1},\cdots ,r_{in}\right\} \end{array}$$ (12)

***Step 5*:** Calculate the Euclidean distance between the evaluation object and the optimal solution and the worst solution, as shown in Eq. (13) and Eq. (14).

$$\begin{array}{c} D_{i}^{+}\text{=}\sqrt{\overset{n}{\underset{j=1}{\sum }}{\left(Q_{i}^{+}-r_{ij}\right)}^{2}},\left(i=1,2,\cdots ,m\right) \end{array}$$ (13)

$$\begin{array}{c} D_{i}^{-}\text{=}\sqrt{\overset{n}{\underset{j=1}{\sum }}{\left(Q_{i}^{-}-r_{ij}\right)}^{2}},\left(i=1,2,\cdots ,m\right) \end{array}$$ (14)

***Step 6*:** Calculate the total comprehensive evaluation result and the specific calculation formula is shown in Eq. (15).

$$\begin{array}{c} C_{i}=\frac{D_{i}^{-}}{\left(D_{i}^{+}+D_{i}^{-}\right)},\left(i=1,2,\cdots ,m\right) \end{array}$$ (15)

## 4. Numerical example

Two case studies are selected in this section, the first one is the case of the Bitcoin dataset and the second one is the case of the BYD stock dataset. The selection of relevant parameters is referred to **Section 3**.

### 4.1 Analysis based on the Bitcoin dataset

#### 4.1.1 Collection and analysis of initial data sets.

**Fig 3:**
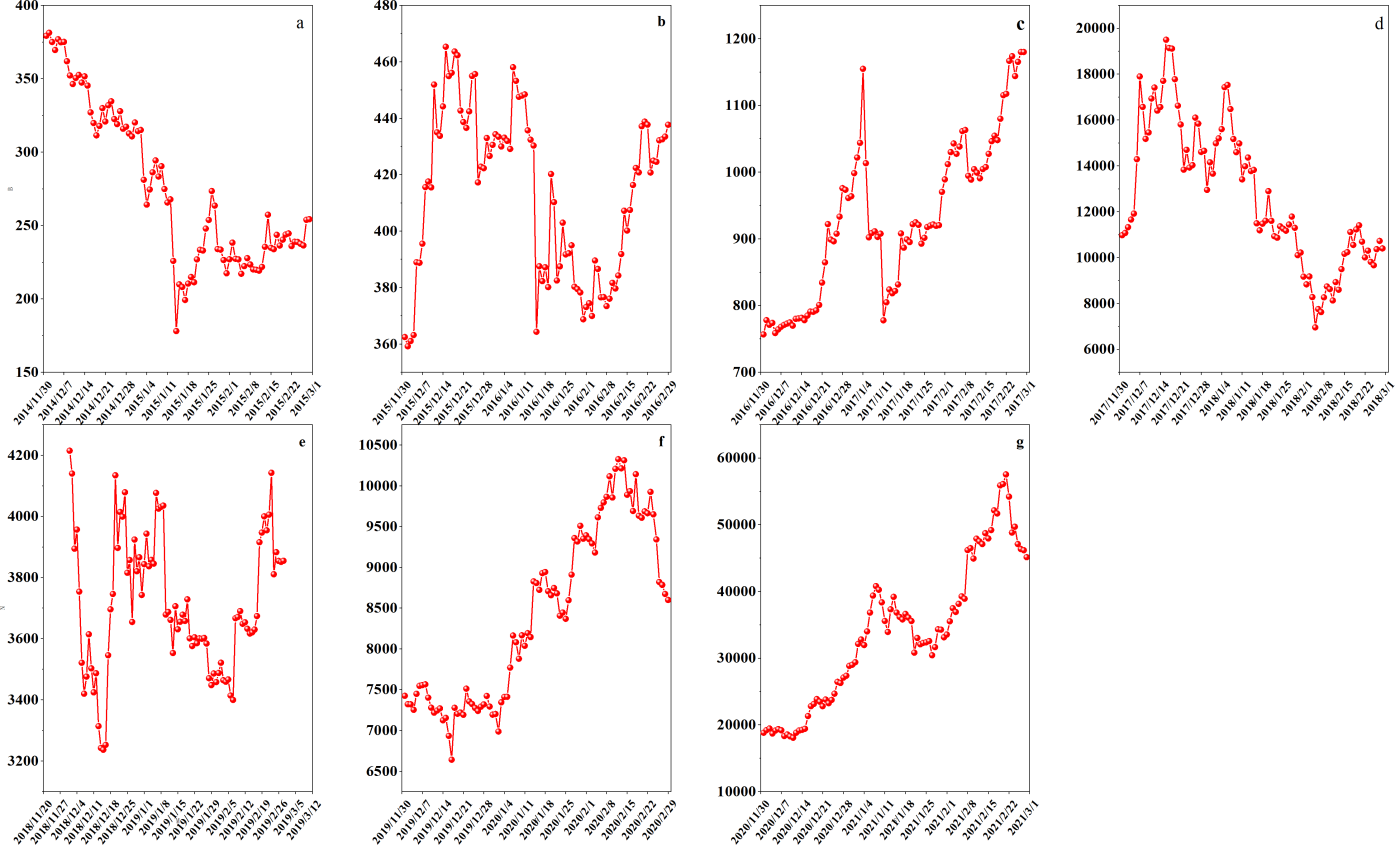
Seven consecutive December-February closing prices.

**Fig 4:**
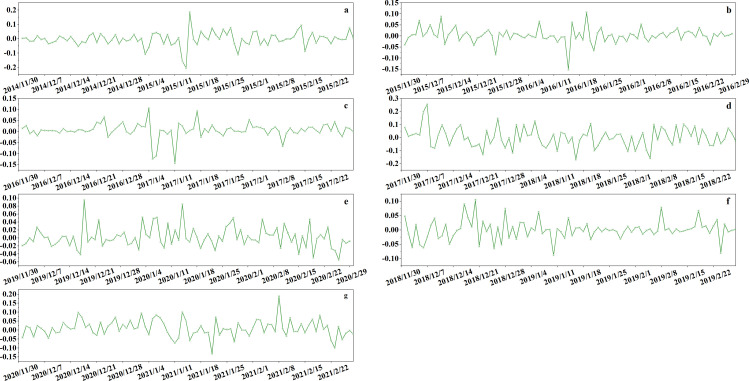
The initial transaction data.

This study uses Bitcoin as a representative example to demonstrate the application of the proposed model on non-customized data assets. Data from 2015 to 2021 was collected to validate the model using Bitcoin as an example. The study examines daily closing prices and gains from December 1 to the end of February each year from 2015 to 2021, as illustrated in Figs 3 and 4. Extensive data analysis was conducted. Fig 3(a) displays daily closing prices from December 1, 2014, to February 28, 2015, and Fig 3(b) from December 1, 2015, to February 29, 2016. Figs 3(c) to 3(f) illustrate daily closing prices from December 1 of each year through February 28 or 29 of the following years: (c) 2016–2017, (d) 2017–2018, (e) 2018–2019, (f) 2019–2020, and (g) 2020–2021. Fig 3(g) displays daily closing prices from December 1, 2020, to February 28, 2021. Examining the December-to-February data reveals a clear trend in Bitcoin’s closing prices during these months across different years. Fig 4 presents the daily changes corresponding to the closing prices shown in Fig 3.

Observing Figs 3 and 4 reveals a clear downward trend from late 2014 to early 2015 and from late 2017 to early 2018, indicating that these periods were not ideal for purchasing Bitcoin. Late 2015 to early 2016 and late 2016 to early 2017 show an upward trend, suggesting these were more favorable times for purchasing Bitcoin. Data from late 2018 to early 2019 shows significant volatility, indicating that this period was less favorable for Bitcoin purchases. The periods from late 2019 to early 2020 and from late 2020 to early 2021 show a downward trend, suggesting uncertainty regarding the suitability for Bitcoin investment. Table 3 shows the calculation results of this study.

#### 4.1.2 Determination of indicator weights.

**Table 3:** Parameter table of indicators of bitcoin.

Primary indicator	Intrinsic Factor					
**Secondary indicator**	**Data source**	**Data scale**	**Data format**	**Data quality**	**Value mining**	**Data governance**
14.12 ~ 15.02	7.5	0.5	0.5	6	8	1.5
15.12 ~ 16.02	6	0.5	0.5	6	8	0.5
16.12 ~ 17.02	5	0.5	1	6.5	8	0.5
17.12 ~ 18.02	4.5	0.5	0.5	6	8	3
18.12 ~ 19.02	7.5	0.5	0.5	6	8	4
19.12 ~ 20.02	2	0.5	1.5	6	5	1.5
20.12 ~ 21.02	5.5	0.5	2	6.5	8	2.5
**Primary indicator**		**External Environmental Factors**				
**Secondary indicator**	**Attribution of assets**	**Technological development**	**User demand**	**Data Ecology**	**Regulatory environment**	**Market development**
14.12 ~ 15.02	2	2.5	5.5	4	4.5	4
15.12 ~ 16.02	2	3	1	2	5.5	9
16.12 ~ 17.02	2	3	4.5	2	5	7.5
17.12 ~ 18.02	2.5	2	4	4	2	4
18.12 ~ 19.02	2.5	2	3	2.5	5.5	1.5
19.12 ~ 20.02	2	2.5	10	3	4	3.5
20.12 ~ 21.02	2	4	4	2	1.5	7.5
**Primary indicator**		**Upstream and downstream assets**		**Interactions between different asset classes**		
**Secondary indicator**	**Social cognition**	**Upstream source data**	**Downstream applications**	**Other non-custom assets**	**Custom Assets**	
14.12 ~ 15.02	1	1	1	0.25	0.25	
15.12 ~ 16.02	4	0.5	1	0.25	0.25	
16.12 ~ 17.02	2.5	0.5	1	0.25	0.25	
17.12 ~ 18.02	4	1	1	1.5	1.5	
18.12 ~ 19.02	2	1.5	1	1	1	
19.12 ~ 20.02	2.5	1.5	1	2.5	1	
20.12 ~ 21.02	2.5	1	0.5	0.25	0.25	

**Fig 5:**
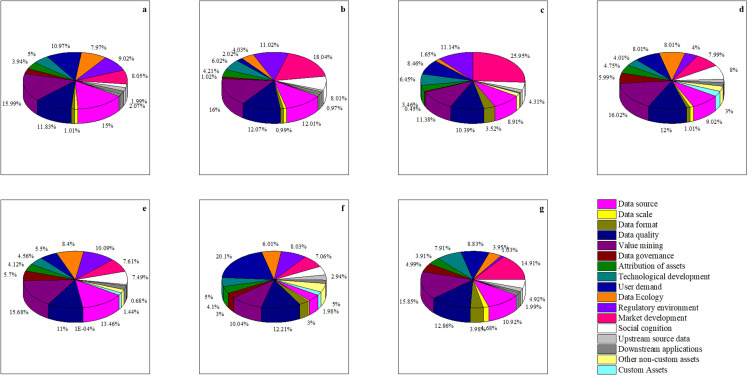
The proportion of each indicator in each year of bitcoin.

After all layers are trained and the accuracy of network training is reached, the final weight matrix obtained is shown in Tables 4–6. The proportion of each indicator in each year is shown in Fig 5.

**Table 4:** Weights of indicator 1 to indicator 6 of bitcoin.

Indicator	Data source	Data scale	Data format	Data quality	Value mining	Data governance
**14.12 ~ 15.2**	0.149973	0.010380	0.010085	0.118331	0.159901	0.029840
**15.12 ~ 16.2**	0.120099	0.009015	0.009946	0.120718	0.160042	0.010161
**16.12 ~ 17.2**	0.089082	0.000001	0.035217	0.103926	0.113805	0.004492
**17.12 ~ 18.2**	0.090246	0.011425	0.010063	0.119963	0.160183	0.059939
**18.12 ~ 19.2**	0.134620	0.000001	0.000001	0.109955	0.156839	0.056975
**19.12 ~ 20.2**	0.040398	0.005268	0.030034	0.122126	0.100376	0.030038
**20.12 ~ 21.2**	0.109187	0.016833	0.039813	0.128563	0.158548	0.049867

**Table 5:** Weights of indicator 7 to indicator 12 of bitcoin.

Indicator	Attribution of assets	Technological development	User demand	Data Ecology	Regulatory environment	Market development
**14.12 ~ 15.2**	0.039435	0.050032	0.109703	0.079731	0.090173	0.080474
**15.12 ~ 16.2**	0.042095	0.060229	0.020179	0.040291	0.110179	0.180440
**16.12 ~ 17.2**	0.034623	0.064479	0.084588	0.016541	0.111385	0.259527
**17.12 ~ 18.2**	0.047543	0.040080	0.080124	0.080064	0.039977	0.079881
**18.12 ~ 19.2**	0.041155	0.045592	0.055020	0.083977	0.100949	0.076136
**19.12 ~ 20.2**	0.041024	0.050025	0.200952	0.060133	0.080284	0.070612
**20.12 ~ 21.2**	0.039083	0.079134	0.088287	0.039510	0.010269	0.149110

**Table 6:** Weights of indicator 13 to indicator 17 of bitcoin.

Indicator	Social cognition	Upstream source data	Downstream applications	Other non-custom assets	Custom Assets
**14.12 ~ 15.2**	0.019867	0.020701	0.021000	0.005071	0.005306
**15.12 ~ 16.2**	0.080101	0.009714	0.017316	0.004908	0.004567
**16.12 ~ 17.2**	0.043098	0.015793	0.005552	0.017892	0.000001
**17.12 ~ 18.2**	0.080044	0.020000	0.020338	0.030088	0.030042
**18.12 ~ 19.2**	0.074866	0.006789	0.022327	0.020351	0.014450
**19.12 ~ 20.2**	0.050132	0.029428	0.019284	0.050049	0.019837
**20.12 ~ 21.2**	0.049204	0.019934	0.012461	0.004901	0.005293

**Fig 6:**
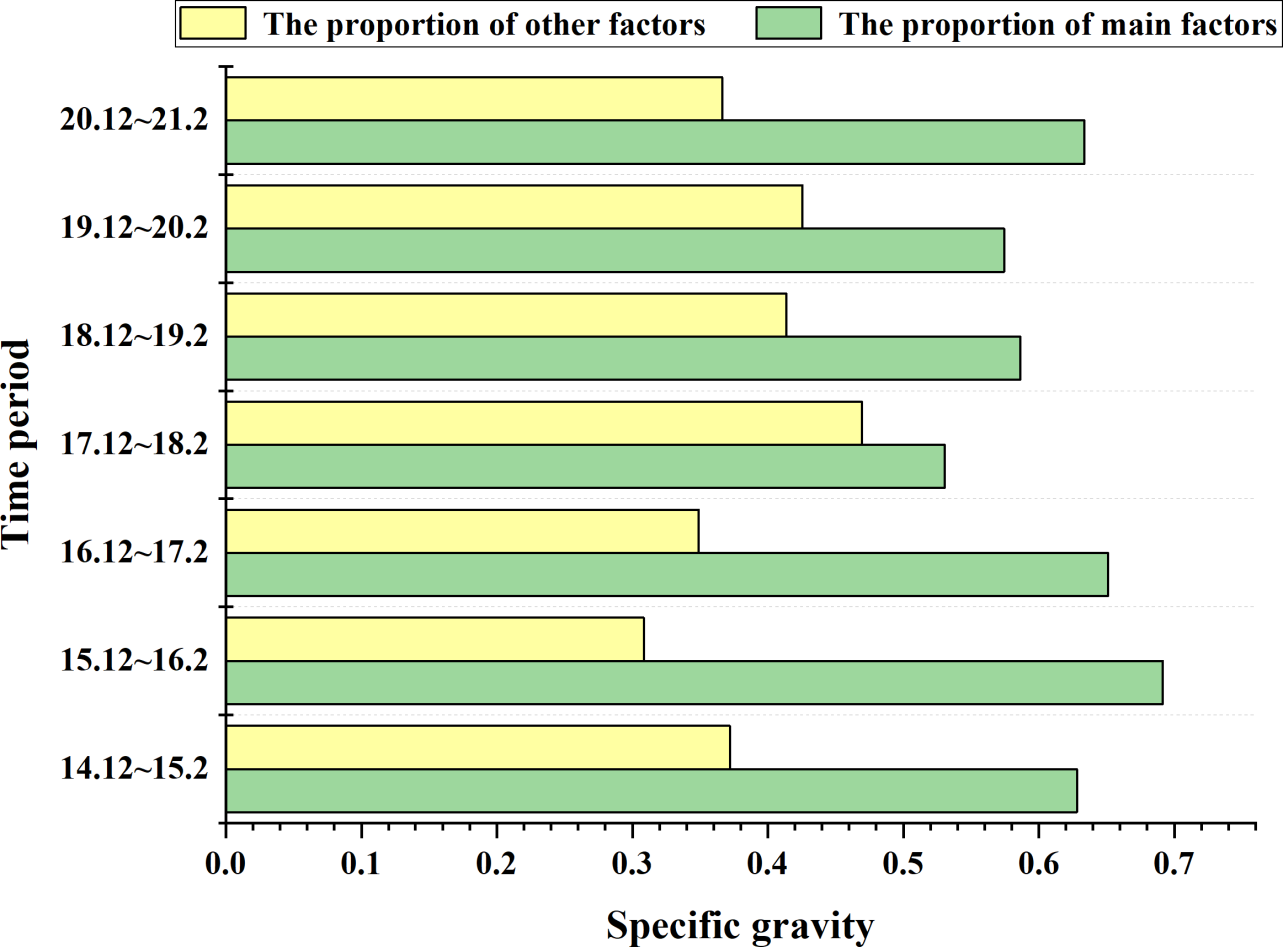
The proportion of five main factors and other factors of bitcoin.

Figs 5(a) through 5(g) display the annual shares of each of the 17 indicators from 2015 to 2021. According to Fig 5(a), the top five factors influencing Bitcoin prices from December 2014 to February 2015 are Data Source, Data Quality, Value Mining, User Demand, and Regulatory Environment. Fig 6 shows that the percentage share of these five key factors during this period is 0.628081. From December 2015 to February 2016, Fig 5(b) indicates that the top five factors impacting Bitcoin prices are Data Source, Data Quality, Value Mining, Regulatory Environment, and Market Development, with a combined percentage share of 0.691478. According to Fig 5(c), the top five factors from December 2016 to February 2017 are Data Source, Data Quality, Value Mining, User Demand, and Market Development, with a total percentage share of 0.650928 as indicated in Fig 6. Fig 5(d) shows that from December 2017 to February 2018, the leading factors influencing Bitcoin are Data Source, Data Quality, Value Mining, User Demand, and Data Ecology, with a share percentage of 0.530580 as indicated in Fig 6. Fig 5(e) shows that from December 2018 to February 2019, the main factors affecting Bitcoin prices are Data Source, Data Quality, Value Mining, Data Ecology, and Regulatory Environment, with a percentage share of 0.586340, as per Fig 6. Fig 5(f) indicates that from December 2019 to February 2020, the leading factors influencing Bitcoin are Data Quality, Value Mining, User Demand, Regulatory Environment, and Market Development, with a share percentage of 0.574350, as shown in Fig 6. According to Fig 5(g), from December 2020 to February 2021, the top factors impacting Bitcoin prices are Data Source, Data Quality, Value Mining, User Demand, and Market Development, with a combined percentage share of 0.633697, as per Fig 6. Analysis of the above weights reveals that Data Quality and Value Mining consistently appear in the top five factors each year. Other key factors impacting Bitcoin include Data Source, User Demand, Regulatory Environment, and Data Ecology.

#### 4.1.3 The establishment of knowledge graph.

The node degree of each indicator is calculated, as shown in Table 7. The node betweenness of each tier-1 indicator is calculated, as shown in Table 8.

**Table 7:** Distribution of node degrees of bitcoin.

Influencing factors	degree	Influencing factors	degree
Intrinsic Factor	10	Data governance	1
External Environmental Factors	8	Attribution of assets	1
Upstream and downstream assets	3	Technological development	1
Interactions between different asset classes	2	User demand	1
External Environmental Factors	1	Data Ecology	1
Upstream and downstream assets	2	Regulatory environment	1
Interactions between different asset classes	3	Market development	1
Data source	1	Social cognition	1
Data scale	1	Upstream source data	1
Data format	1	Downstream applications	1
Data quality	1	Other non-custom assets	1
Value mining	1	Custom Assets	1

**Table 8:** Distribution of node betweenness of bitcoin.

Influencing factors	node betweenness
Intrinsic Factor	0.498024
External Environmental Factors	0.399209
Upstream and downstream assets	0.270751
Interactions between different asset classes	0.326087

**Fig 7:**
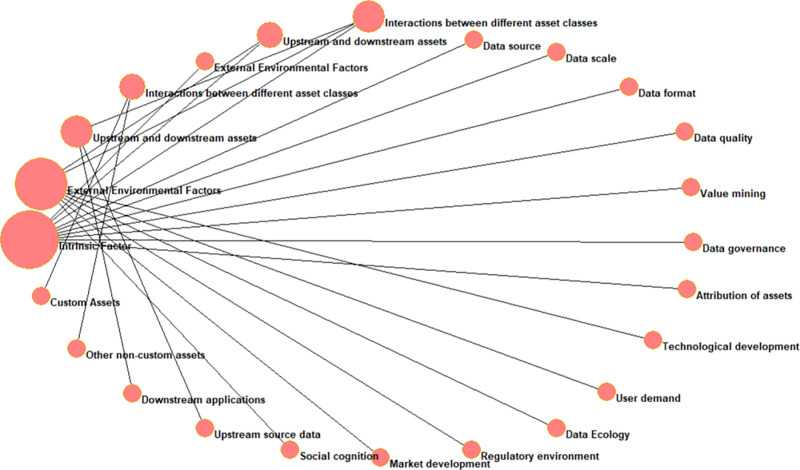
Knowledge graph based on degrees.

As can be seen from Table 7, the node degree of the four level 1 indicators is large, with intrinsic factor having the largest node degree, implying that intrinsic factor can influence the most other factors. From Table 8, it can be seen that the node median of intrinsic factor is the largest, implying that intrinsic factor has more influence. According to the node degree and node median, we used Pajek software to draw a knowledge graph diagram, as shown in Fig 7. Fig 7 represents the knowledge graph constructed from the correlations between the four primary indicators and the seventeen secondary indicators, which are constructed based on the node degree. If there is a connection between two indicators, then there is a link between the two indicators, and if there is no relationship between the two indicators, then there is no link between the two indicators. In addition, the size of the area of the circle is related to the importance of the indicator, the larger the area, the more important the indicator is. According to Fig 7, Intrinsic Factor is the most important because there are 10 indicators connected to it.

#### 4.1.4 Results of Information Entropy-TOPSIS Modeling.

Combined with the weights of the indicators calculated in 4.2, the information entropy and information entropy weights of each indicator are calculated, as shown in Table 8. As can be seen from Table 9, for Bitcoin, among the 17 metrics, Data quality has the largest information entropy and Data scale has the smallest information entropy. The Euclidean distance between the evaluation object and the optimal solution and the worst solution of each indicator, as shown in Table 10. The total comprehensive evaluation result under each evaluation stage was calculated, as shown in Table 11.

**Table 9:** Entropy of Information and their weights of bitcoin.

Indicator	Entropy of Information	Weights of information entropy
**Data source**	0.970706	0.022919
**Data scale**	0.794665	0.160650
**Data format**	0.834494	0.129488
**Data quality**	0.998920	0.000845
**Value mining**	0.992536	0.005840
**Data governance**	0.893134	0.083609
**Attribution of assets**	0.998010	0.001557
**Technological development**	0.987867	0.009493
**User demand**	0.918983	0.063386
**Data Ecology**	0.949222	0.039728
**Regulatory environment**	0.929611	0.055071
**Market development**	0.936111	0.049985
**Social cognition**	0.961371	0.030222
**Upstream source data**	0.956267	0.034216
**Downstream applications**	0.967171	0.025685
**Other non-custom assets**	0.836541	0.127887
**Custom Assets**	0.796237	0.159420

**Table 10:** The Euclidean distance between the evaluation object of bitcoin.

Ideal solution	Negative Ideal Solution
0.02211	0.011008
0.031223	0.011781
0.046352	0.016378
0.013591	0.011443
0.016500	0.010932
0.043455	0.016504
0.0238196	0.012176

**Table 11:** The total comprehensive evaluation result of bitcoin.

Time	Aggregate rating	Ranking
2014.12.01 ~ 2015.02.28	0.332303	4
2015.12.01 ~ 2016.02.29	0.273954	6
2016.12.01 ~ 2017.02.28	0.261092	7
2017.12.01 ~ 2018.02.28	0.457100	1
2018.12.01 ~ 2019.02.28	0.398512	2
2019.12.01 ~ 2020.02.29	0.275255	5
2020.12.01 ~ 2021.02.28	0.338260	3

Table 11 presents the final evaluation results and rankings for the seven time periods. The lower the ranking, the less suitable it is to purchase Bitcoin, based on the indicators used in our model. A closer look at the results reveals some key insights into Bitcoin’s market behavior during these periods.

2017.12.01 ~ 2018.02.28 ranks first with the highest final evaluation index of 0.4571. This period marks a significant upward trend in Bitcoin’s value, reaching its lowest point before the rise to its peak in early 2018. The model’s evaluation indicates this is the most optimal time to buy Bitcoin, as the indicators suggest a strong potential for value appreciation. This finding aligns well with the actual market data, confirming that purchasing Bitcoin during this time period would have been a favorable decision. For businesses or investors, this implies that the framework could serve as a valuable tool for identifying profitable investment windows by assessing the timing of upward trends based on multiple indicators.

2016.12.01 ~ 2017.02.28, on the other hand, ranks the lowest with a final evaluation score of 0.2611. During this time, Bitcoin had already risen significantly and was nearing its yearly maximum. As a result, there was a high probability of a price correction, making this the least favorable period to purchase Bitcoin. Again, the model’s prediction aligns with the actual market movement, where Bitcoin’s value began to decrease after this period. For businesses, this demonstrates the potential of the model to avoid investments during market peaks, thereby preventing potential losses from overvalued assets.

Overall, when comparing the model’s calculated results with actual Bitcoin market trends, it becomes evident that the model provides a reliable assessment of Bitcoin’s purchasing potential. This reinforces the model’s ability to forecast favorable investment periods and avoid risky investments. For businesses, this framework can be a powerful tool to inform decision-making processes by providing data-driven insights into market timing, helping to minimize risks and optimize returns.

### 4.2 Analysis based on the stock dataset of BYD Company

**Fig 8:**
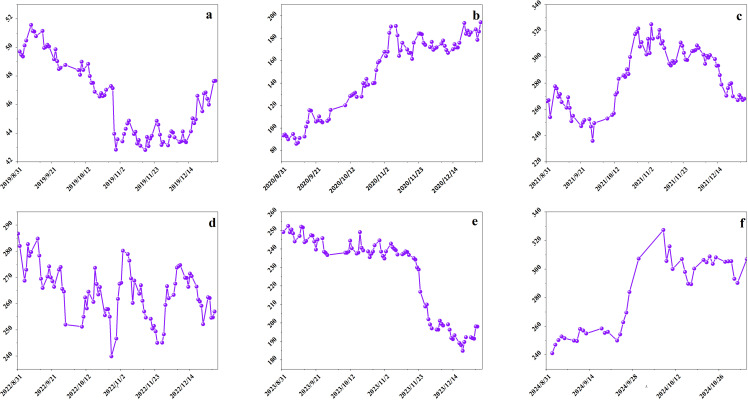
Closing price of BYD Stock.

**Fig 9:**
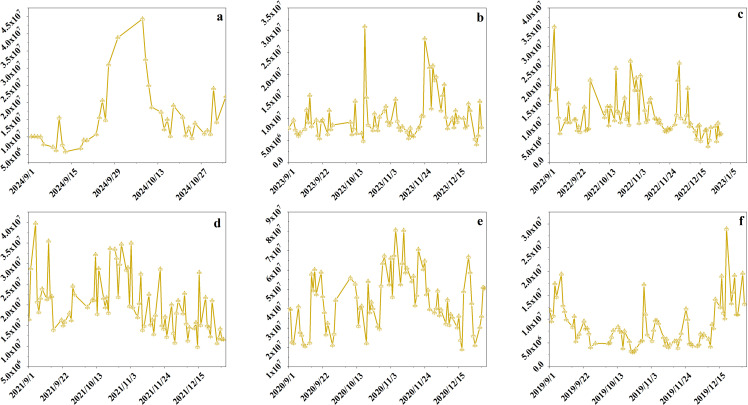
Volume of BYD Stock.

The second study case is BYD stock, and the relevant parameters are selected with reference to 4.1. Figs 8 and 9 show the changes in the closing price and volume of BYD stock from 2019 to 2024. Tables 12–14 show the weights of each indicator for each year of BYD stock calculated by the neural network based weighting method of this paper.

**Table 12:** Weights of indicator 1 to indicator 6 of BYD Stock.

Indicator	Data source	Data scale	Data format	Data quality	Value mining	Data governance
19.09 ~ 19.12	0.104618	0.031541	0.010086	0.116811	0.088794	0.000001
20.09 ~ 20.12	0.109128	0.031336	0.019876	0.113592	0.166003	0.003493
21.09 ~ 21.12	0.100387	0.031419	0.019614	0.124832	0.147635	0.012454
22.09 ~ 22.12	0.120763	0.025663	0.020791	0.066873	0.112818	0.034145
23.09 ~ 23.12	0.119250	0.028603	0.019601	0.104758	0.089940	0.071973
24.09 ~ 24.11	0.123829	0.022423	0.021391	0.108085	0.043163	0.022509

**Table 13:** Weights of indicator 7 to indicator 12 of BYD Stock.

Indicator	Attribution of assets	Technological development	User demand	Data Ecology	Regulatory environment	Market development
19.09 ~ 19.12	0.057704	0.099747	0.035796	0.071954	0.131188	0.138340
20.09 ~ 20.12	0.036839	0.053428	0.055774	0.034037	0.109215	0.153910
21.09 ~ 21.12	0.034254	0.064074	0.073370	0.060068	0.095846	0.145772
22.09 ~ 22.12	0.023402	0.177624	0.054780	0.120104	0.098292	0.061809
23.09 ~ 23.12	0.072766	0.136858	0.036501	0.046008	0.083712	0.060169
24.09 ~ 24.11	0.037803	0.206901	0.043145	0.142700	0.055724	0.100208

**Table 14:** Weights of indicator 13 to indicator 17 BYD Stock.

Indicators	Social cognition	Upstream source data	Downstream applications	Other non-custom assets	Custom Assets
19.09 ~ 19.12	0.063889	0.014878	0.016024	0.002729	0.015899
20.09 ~ 20.12	0.064897	0.016078	0.018454	0.007921	0.006020
21.09 ~ 21.12	0.037877	0.009400	0.025320	0.013222	0.004454
22.09 ~ 22.12	0.044312	0.010789	0.007602	0.000001	0.020235
23.09 ~ 23.12	0.039241	0.034201	0.029792	0.016968	0.009659
24.09 ~ 24.11	0.014425	0.011433	0.019514	0.013212	0.013536

**Fig 10:**
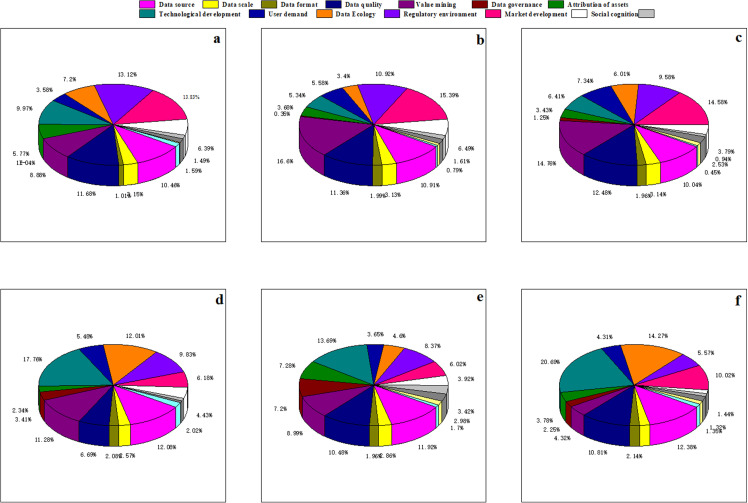
The proportion of each indicator in each year of BYD Stock.

**Fig 11:**
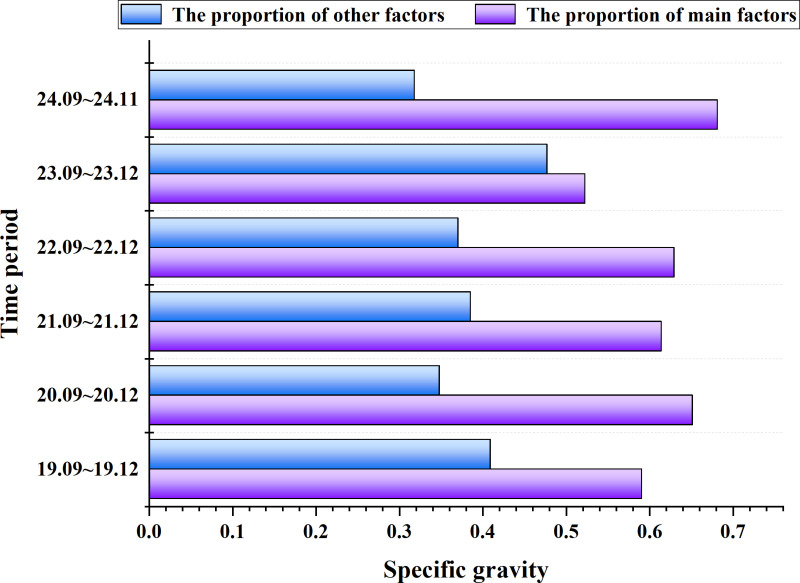
The proportion of five main factors and other factors of BYD Stock.

Fig 10(a).Fig 5(f) correspond to the annual share of each of the 17 metrics for each of the years 2019 through 2024, respectively. From Fig 10(a), from December 2019 to February 202020, the top five factors affecting the price of Bitcoin are, in order, Data source, Data quality, Technological development, Regulatory environment, and Market development.According to Fig 6, the share of the five key factors in this period is 0.590705. From Fig 10(b), from December 2020 to February 2021, the top five factors affecting the price of Bitcoin are, in order, Data source, Data quality, Value mining, Regulatory environment and Market development. according to Fig 6, the percentage share of the five key factors is 0.590705 for this period. The percentage of the five key factors is 0.651847. From Fig 10(c), from December 2021 to February 2022, the top five factors affecting the price of Bitcoin are Data source, Data quality, Value mining, Regulatory environment, and Market development, in order of magnitude. According to Fig 6, the percentage of the five key factors in this period is 0.614474. According to Fig 10(d), from December 2022 to February 2023, the top five factors affecting the price of Bitcoin are Data source, Value mining, Technological development, Data Ecology, and Regulatory environment in order. Data source, Value mining, Technological development, Data Ecology, and Regulatory environment. According to Fig 6, it can be seen that the percentage of the five key factors in this period is 0.629600. From Fig 10(e), from December 2022 to February 2023, the top five factors affecting the price of Bitcoin are, in order, Data source, Data quality, Value mining, Technological development, Data governance and Technological development.According to Fig 6, the percentage of the five key factors in this period is 0.522779. From Fig 10(f), from December 2022 to February 2023, the top five factors affecting the price of Bitcoin are Data source, Data quality, Technological development, Data Ecology, and Market development, in order of magnitude. percentage of the five key factors in this period is 0.681724 The percentage of the five key factors in this period is 0.681724 (Fig 11).

Entropy of Information and their weights BYD Stock are shown in Table 15. The Euclidean distance between the evaluation object and the optimal solution and the worst solution of each indicator is calculated, as shown in Table 16. The total comprehensive evaluation result under each evaluation stage was calculated, as shown in Table 17.

**Table 15:** Entropy of Information and their weights BYD Stock.

Indicator	Entropy of Information	Weights of information entropy
**Data source**	0.998312	0.001720
**Data scale**	0.995809	0.004268
**Data format**	0.986161	0.014094
**Data quality**	0.990569	0.009606
**Value mining**	0.958120	0.042653
**Data governance**	0.713752	0.291530
**Attribution of assets**	0.96210	0.038604
**Technological development**	0.939843	0.061267
**User demand**	0.981251	0.019094
**Data Ecology**	0.932882	0.068357
**Regulatory environment**	0.983021	0.017293
**Market development**	0.963673	0.036997
**Social cognition**	0.952623	0.048251
**Upstream source data**	0.935974	0.065207
**Downstream applications**	0.960675	0.040060
**Other non-custom assets**	0.828733	0.174428
**Custom Assets**	0.9346245	0.066582

**Table 16:** The Euclidean distance between the evaluation object BYD Stock.

Ideal solution	Negative Ideal Solution
0.018939	0.011232
0.022762	0.010630
0.018933	0.011096
0.036711	0.018045
0.076071	0.023621
0.043851	0.01790

**Table 17:** The total comprehensive evaluation result BYD Stock.

Time	Aggregate rating	Ranking
19.09 ~ 19.12	0.372291	1
20.09 ~ 20.12	0.318335	4
21.09 ~ 21.12	0.369519	2
22.09 ~ 22.12	0.329550	3
23.09 ~ 23.12	0.236941	6
24.09 ~ 24.11	0.289847	5

Table 17 displays the total comprehensive evaluation results and rankings for BYD stock over several time periods. The lower the ranking, the less suitable it is to purchase BYD stock, as per the model’s evaluation. The results provide meaningful insights into the stock’s performance, which can be compared to its actual market trends.

2019.09 ~ 2019.12 ranks first with an aggregate rating of 0.372291, indicating this is the most suitable time to buy BYD stock. During this period, BYD stock shows a clear upward trend in its closing price and trading volume, which is consistent with the model's evaluation. The calculated ranking reflects the favorable market conditions for purchasing at this time, confirming that this period would have been an optimal investment opportunity. This highlights the usefulness of the framework in identifying profitable windows for investment by analyzing key performance indicators.

2023.09 ~ 2023.12 ranks the lowest with an aggregate rating of 0.236941. This time period corresponds with a phase of downward movement in both the closing price and trading volume of BYD stock. The model’s ranking aligns with the actual market trend, as this was a less favorable time to invest in the stock due to its declining performance. The results suggest that investors should avoid purchasing during this period, which would have been a riskier investment choice. This emphasizes the model’s capacity to steer investors away from unfavorable market conditions.

The analysis of the BYD stock results demonstrates that the model effectively aligns with the actual stock trends over the periods considered. By comparing the rankings and evaluation scores with real market data, it is evident that the model provides accurate predictions of the optimal and least favorable times to invest. This reinforces the value of the methodology, as it helps investors and businesses avoid potential losses and identify profitable opportunities. The results highlight how this framework can be used to make more data-driven and informed investment decisions, optimizing returns while minimizing risks in dynamic markets.

## 5. Conclusion

This paper developed a comprehensive evaluation framework using the Information Entropy-TOPSIS model to assess non-customized data assets, demonstrated through the analysis of Bitcoin and BYD stock. The results show that the model accurately reflects market trends, successfully identifying the optimal investment periods. Specifically, the highest ranking for Bitcoin occurred in the 2017.12–2018.02 period, which was marked by a significant upward trend in price, while the lowest ranking occurred during 2016.12–2017.02, when prices were near their peak and most likely to fall. Similarly, for BYD stock, the most favorable period was 19.09–19.12, aligning with an upward market trend, while the least favorable was 23.09–23.12, corresponding with a market downturn.

The practical implications of these findings are significant for businesses and investors. The model offers a robust framework for determining optimal investment timing, minimizing risk, and enhancing returns by aligning investment decisions with market trends. This methodology can be particularly useful for businesses seeking to make data-driven, strategic decisions in volatile markets. By quantifying asset value based on real-time data, companies can avoid potential losses and identify lucrative opportunities.

Looking ahead, future research could enhance this model by incorporating additional variables, such as market sentiment or macroeconomic indicators, to provide more comprehensive evaluations. Further exploration into machine learning integration could allow for dynamic adjustments to the model’s parameters based on real-time market conditions, offering even more precise decision-making support for businesses and investors in the future.
